# Emergency medical services evaluations for chest pain during first COVID-19 lockdown in Hollands-Midden, the Netherlands

**DOI:** 10.1007/s12471-021-01545-y

**Published:** 2021-02-18

**Authors:** E. R. de Koning, M. J. Boogers, J. Bosch, M. de Visser, M. J. Schalij, S. L. M. A. Beeres

**Affiliations:** 1 Department of Cardiology, Leiden University Medical Centre, Leiden, The Netherlands; 2 Research and Development, Regional Ambulance Service Hollands-Midden, Leiden, The Netherlands

**Keywords:** COVID-19, Chest pain, STEMI, OHCA

## Abstract

**Objective:**

To assess whether the COVID-19 lockdown in 2020 had negative indirect health effects, as people seem to have been reluctant to seek medical care.

**Methods:**

All emergency medical services (EMS) transports for chest pain or out-of-hospital cardiac arrest (OHCA) in the Dutch region Hollands-Midden (population served > 800,000) were evaluated during the initial 6 weeks of the COVID-19 lockdown and during the same time period in 2019. The primary endpoint was the number of evaluated chest pain patients in both cohorts. In addition, the number of EMS evaluations of ST-elevation myocardial infarction (STEMI) and OHCA were assessed.

**Results:**

During the COVID-19 lockdown period, the EMS evaluated 927 chest pain patients (49% male, age 62 ± 17 years) compared with 1041 patients (51% male, 63 ± 17 years) in the same period in 2019, which corresponded with a significant relative risk (RR) reduction of 0.88 (95% confidence interval (CI) 0.81–0.96). Similarly, there was a significant reduction in the number of STEMI patients (RR 0.52, 95% CI 0.32–0.85), the incidence of OHCA remained unchanged (RR 1.23, 95% CI 0.83–1.83).

**Conclusion:**

During the first COVID-19 lockdown, there was a significant reduction in the number of patients with chest pain or STEMI evaluated by the EMS, while the incidence of OHCA remained similar. Although the reason for the decrease in chest pain and STEMI consultations is not entirely clear, more attention should be paid to the importance of contacting the EMS in case of suspected cardiac symptoms in possible future lockdowns.

**Supplementary Information:**

The online version of this article (10.1007/s12471-021-01545-y) contains supplementary material, which is available to authorized users.

## What’s new?

- COVID-19 lockdowns have led to fewer admissions for ST-elevation myocardial infarction (STEMI) and an increased incidence of out-of-hospital cardiac arrest (OHCA) in heavily affected areas worldwide, suggesting people’s reluctance to contact medical professionals when they experience chest pain.
- While previous reports have focused on the incidence of STEMI or OHCA, the total number of patients with chest pain has not been evaluated so far. This is the first study investigating the number of chest pain patients evaluated by the emergency medical services during a COVID-19 lockdown and the first study on the incidence of chest pain in the Netherlands.
- This study showed a significant decrease in chest pain and STEMI consultations. Therefore, patients experiencing chest pain should be actively encouraged to contact medical professionals during a COVID-19 lockdown.

## Introduction

The rapid spread of the severe acute respiratory syndrome coronavirus 2 (SARS-CoV-2), responsible for coronavirus disease 2019 (COVID-19), urged the Dutch government to announce a national lockdown, starting 16 March 2020. Apart from social distancing, people were encouraged to stay at home as much as possible and schools were closed. These measures were effective in controlling the spread of the virus and reduced the pressure on the Dutch healthcare system.

There is growing interest in the indirect health effects of the COVID-19 lockdown period. On the one hand, improved air quality and reduced work-related stress might have been beneficial. On the other hand, fear of contracting the virus made people cancel, postpone or limit even urgent medical treatments, potentially resulting in life-threatening situations [1]. Indeed, during the COVID-19 lockdown period, a significant increase in out-of-hospital cardiac arrest (OHCA) has been observed in Italy and France [2–4].

This finding can be partly attributed to the complications of COVID-19 but may also be related to late presentations of ST-elevation myocardial infarction (STEMI) patients with electrical or mechanical complications. Consistently, a decrease in STEMI admissions in the COVID-19 pandemic period has been observed in Italy, France, Spain and the USA [5–11]. Tan et al. analysed cardiac catheterisations in California, USA and reported that the drop in STEMI admissions was paralleled by a decrease in coronary catheterisations for patients with unstable angina or non–ST-elevation myocardial infarction [12]. However, it remains to be determined whether chest pain patients across the board were reluctant to seek acute medical care during the COVID-19 lockdown.

The aim of the current study was to investigate the number of chest pain patients evaluated by the emergency medical services (EMS) during the COVID-19 lockdown in the Netherlands in 2020. Simultaneously, EMS alerts for STEMI and OHCA were assessed. These data may attribute to the understanding of the indirect health effects of the COVID-19 lockdown and may contribute to forthcoming guidelines for acute care management during possible future lockdowns.

## Methods

The AmbuSuite database (Topicus, the Netherlands) contains data of all ambulance transports performed by the Regional Ambulance Service Hollands-Midden (RAVHM), the EMS of the Dutch security region Hollands-Midden, which has over 800,000 inhabitants. All data in the AmbuSuite database are collected prospectively by the paramedics on the ambulance and include patient data (medical history, main complaint, other symptoms, vital parameters, electrocardiogram and working diagnosis), as well as data regarding the ambulance ride (dispatch time, time of arrival at the scene, time of arrival at the hospital, and address of scene and hospital). The decision to dispatch an ambulance to a patient is made in the regional control centre and is based on the Advanced Medical Priority Dispatch System, Professional Quality Assurance, which was similar in 2019 and 2020 [13].

All patients for whom an ambulance was dispatched by the RAVHM during the initial 6 weeks of the first COVID-19 lockdown period in the Netherlands (week 12–17 in 2020) were eligible for inclusion in the current study. Inclusion criteria were: (1) age over 18 years, and (2) ambulance ride because of a main complaint of chest pain or nontraumatic OHCA.

If the paramedics suspected a STEMI, this diagnosis was verified in the central percutaneous coronary intervention centre in the region. A nontraumatic OHCA was defined as any cardiac arrest after exclusion of cases with obvious accidental causes, irrespective of whether resuscitation was attempted or not. To determine the cause of OHCA, the EMS reports of all OHCA cases were analysed on a case-by-case basis by two experienced reviewers (EdK and SB). OHCA cases were divided in shockable rhythm and nonshockable rhythm on arrival by the EMS, with the latter category further subdivided in the following causal categories: cardiac aetiology, related to COVID-19 and unknown aetiology.

For the control group, the same inclusion criteria and definitions were applied to all patients for whom an ambulance was dispatched by the RAVHM in week 12–17 in 2019.

This study complied with the Declaration of Helsinki. The institutional medical ethics committee approved the study protocol (G20.111) and waived the need for individual informed consent.

### Statistical analyses

Categorical data were compared using the chi-squared test and are presented herein as number with percentage. Continuous data were compared with a one-way ANOVA or Kruskal-Wallis test and are presented as mean ± standard deviation. The incidence of chest pain, STEMI and OHCA in the COVID-19 lockdown period were compared with the incidence in the same period in 2019. Incidence rates and relative risk (RR) were estimated based on data on the regional population in 2019 and 2020 from Statistics Netherlands (www.cbs.nl) and compared using the chi-squared test.

The data were analysed using R version 3.6.2. *P*‑values < 0.05 were considered statistically significant. All data were coded and anonymised.

## Results

During the first Dutch COVID-19 lockdown period in 2020, the EMS evaluated 927 chest pain patients, compared with 1041 patients during the same period in 2019. As shown in Tab. 1, the characteristics of these patients did not differ between both time periods. In particular, gender and age were similar, as well as haemodynamic parameters.

**Table 1:** Characteristics of chest pain patients evaluated during COVID-19 lockdown in 2020 and during same time period in 2019

Variable	Lockdown (2020) (*n* = 927)	2019 (*n* = 1041)	*P*‑value
Male	455 (49)	534 (51)	0.529
Age, years	62 ± 17	63 ± 17	0.184
Known coronary disease	215 (23)	224 (22)	0.403
Heart rate, bpm	86 ± 28	86 ± 29	0.415
Systolic blood pressure, mmHg	152 ± 31	150 ± 31	0.133
Diastolic blood pressure, mmHg	88 ± 19	88 ± 18	0.487
Time from dispatch to patient, min	8.0 ± 3.7	8.1 ± 3.9	0.536
Time from dispatch to hospital, min	52.0 ± 15.2	47.5 ± 13.9	<0.001

Data are *n* (%), or mean ± standard deviation

*COVID-19* coronavirus disease 2019, *bpm* beats per minute

**Fig. 1:**
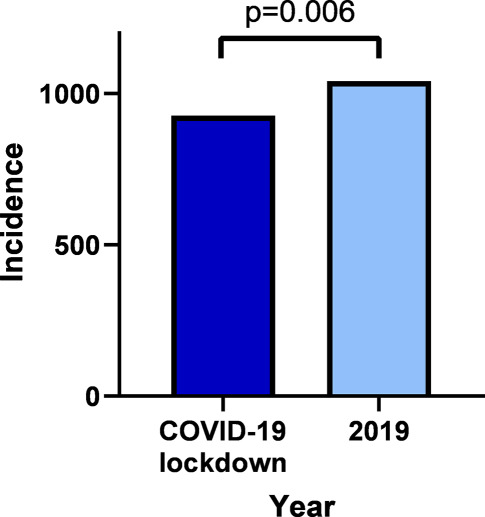
Incidence of chest pain during COVID-19 lockdown period in 2020 and during same time period in 2019

As illustrated in Fig. 1, the incidence of chest pain—defined as the number of chest pain patients evaluated by the EMS divided by the total number of inhabitants in the EMS region—was lower during the COVID-19 lockdown period (927/809,104) than during the same period in 2019 (1041/802,325). This resulted in a significant RR reduction in the incidence of chest pain in the COVID-19 lockdown period of 0.88 (95% confidence interval (CI) 0.81–0.96, *p* = 0.006).

**Fig. 2:**
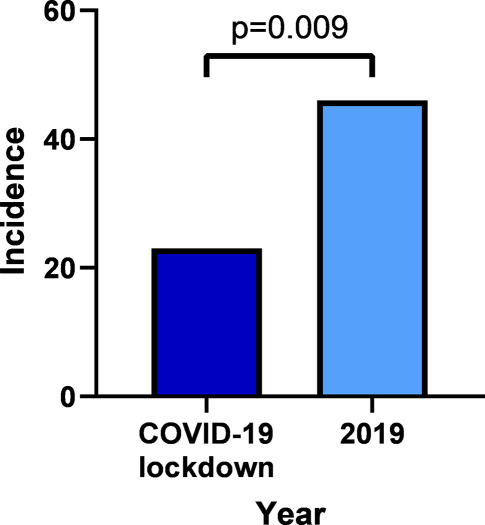
Incidence of ST-elevation myocardial infarction during COVID-19 lockdown period in 2020 and during same time period in 2019

Tab. 2 displays the characteristics of STEMI patients in the COVID-19 lockdown period (*n* = 23) and in the same period in 2019 (*n* = 46). The incidence of STEMI—defined as the number of STEMI patients divided by the total number of inhabitants in the EMS region—was lower during the COVID-19 lockdown period (23/809,104) than during the same period in 2019 (46/802,325). Accordingly, during the COVID-19 lockdown period, there was a significant RR reduction in STEMI incidence of 0.52 (95% CI 0.32–0.85, *p* = 0.009) compared with the same period in 2019 (Fig. 2).

**Table 2:** Characteristics of STEMI patients evaluated during COVID-19 lockdown in 2020 and during same time period in 2019

Variable	Lockdown (2020) (*n* = 23)	2019 (*n* = 46)	*P*‑value
Male	20 (87)	39 (85)	1
Age, years	62 ± 10	64 ± 12	0.567
Known coronary disease	18 (78)	41 (89)	0.283
Heart rate, bpm	76 ± 38	77 ± 32	0.945
Systolic blood pressure, mmHg	140 ± 26	138 ± 46	0.866
Diastolic blood pressure, mmHg	86 ± 20	83 ± 28	0.614
Time from dispatch to patient, min	6.6 ± 2.6	7.5 ± 4.1	0.313
Time from dispatch to hospital, min	47.4 ± 12.8	48.0 ± 11.1	0.856

Data are *n* (%), or mean ± standard deviation

*STEMI* ST-elevation myocardial infarction, *COVID-19* coronavirus disease 2019

The characteristics of OHCA patients in the COVID-19 lockdown period (*n* = 56) and in the same period in 2019 (*n* = 45) are shown in Tab. 3. Both groups were comparable regarding gender, mean age and previously known coronary artery disease. Analysis of the EMS reports revealed a trend towards a different cause of OHCA in the COVID-19 lockdown period compared with the same period in 2019 (*p* = 0.05). In particular, a shockable rhythm upon arrival by the EMS was found in 15 patients (27%) in the COVID-19 lockdown period and in 18 patients (40%) in the same period in 2019; a cardiac aetiology was found in 10 patients (18%) and in 4 patients (9%), respectively. During the COVID-19 lockdown period, COVID-19 was the probable cause of OHCA in 6 patients (11%).

**Table 3:** Characteristics of OHCA patients evaluated during COVID-19 lockdown in 2020 and during same time period in 2019

Variable	Lockdown (2020) (*n* = 56)	2019 (*n* = 45)	*P*‑value
Male	32 (57)	31 (69)	0.086
Age, years	70 ± 14	70 ± 12	0.906
Known coronary disease	15 (62)	14 (58)	1
*OHCA details*			
– Shockable rhythm	15 (27)	18 (40)	0.585
– Nonshockable rhythm, cardiac aetiology	10 (18)	4 (9)	0.125
– Nonshockable rhythm, COVID-19	6 (11)	0	NA
– Nonshockable rhythm, unknown aetiology	25 (44)	23 (51)	0.795
Time from dispatch to patient, min	7.1 ± 3.2	6.0 ± 3.1	0.088
Time from dispatch to hospital, min	48.5 ± 19.1	42.2 ± 11.7	0.207

Data are *n* (%), or mean ± standard deviation

*OHCA* out-of-hospital cardiac arrest, *COVID-19* coronavirus disease 2019, *NA* not applicable

**Fig. 3:**
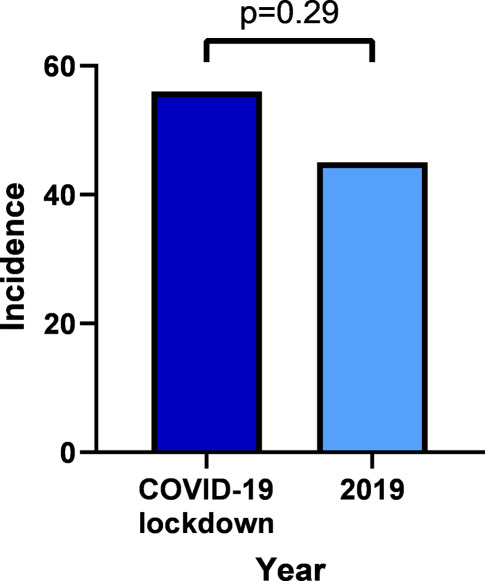
Incidence of out-of-hospital cardiac arrest during COVID-19 lockdown period in 2020 and during same time period in 2019

The incidence of OHCA—defined as the number of OHCA patients divided by the total number of inhabitants in the EMS region—was 56/809,104 during the COVID-19 lockdown period and 45/802,325 during the same period in 2019 (RR 1.23, 95% CI 0.83–1.83, *p* = 0.29; Fig. 3).

## Discussion

The main finding of this study was that the number of patients evaluated by the EMS because of chest pain was lower during the COVID-19 lockdown than during the same period in 2019. This was paralleled by a reduction in the incidence of STEMI, while the incidence of OHCA remained unchanged.

Beyond the harmful effects of COVID-19 on the respiratory and cardiovascular system [14], there have been major concerns about the indirect negative health effects of the COVID-19 lockdown. Fear of being infected when alerting the EMS or attending the hospital, made people cancel, postpone or limit medical treatments, potentially resulting in worse outcomes. To better understand the indirect health effects of the COVID-19 pandemic, previous reports have focused on STEMI and OHCA; however, it is still unknown whether patients with all types of chest pain were reluctant to request acute medical care during a COVID-19 lockdown. In this perspective, the current study provides novel insights, as it showed significantly less chest pain patients were evaluated by the EMS during the Dutch lockdown in 2020 than in the same period in 2019. The current findings are in line with those of Tan et al., which showed a decline of 26% in the number of patients with all types of acute coronary syndrome undergoing cardiac catheterisation in a single centre in California during the COVID-19 pandemic [12].

As part of the broad spectrum of chest pain evaluations, we also evaluated EMS alerts for STEMI and showed a significantly reduced number of STEMIs during the COVID-19 lockdown compared with the same period in 2019. This is in line with the previously reported sharp decrease in STEMIs during the COVID-19 lockdown [5–11]. De Rosa et al. reported a clear reduction in acute myocardial infarction in Italy, where the number of hospitalisations halved compared with the previous year, with a 26.5% reduction in STEMI diagnoses [5]. Garcia et al. demonstrated a decrease of 38% in STEMI referrals for nine centres in the USA during the COVID-19 period [11].

Our reported decline in the number of chest pain patients as well as STEMI patients admitted to hospitals during the COVID-19 lockdown period may be conceptually attributed to different pathophysiological, environmental and behavioural factors. One can hypothesise that less vigorous physical exercise or reduced physiological stress during a lockdown can result in fewer coronary plaque ruptures. Intense physical exertion reportedly leads to enhanced thrombogenic tendency, increased blood viscosity and increased propensity for thrombocyte aggregation and thereby results in an elevated risk of acute myocardial infarction [15].

Furthermore, changes in the environment may play a role in lowering the incidence of cardiovascular morbidity and mortality, since the COVID-19 lockdown has been associated with a dramatic decline in air pollution [16, 17]. Reduced exposure to air pollution due to less traffic and industrial activities during a lockdown, may lead to fewer cardiovascular events. Studies have shown that even short-term exposure to elevated air pollution or traffic exposure is positively associated with elevated risk of myocardial infarction. The underlying pathophysiology is not completely understood, but fine particulate matter–induced inflammation, oxidative stress, and vascular dysfunction are all named as possible contributing factors [18]. Several reports have also shown more hospital admissions for ischaemic heart disease, with short-term elevation of inhalable or particulate matter air pollution [19].

Last, but most probably not least, behavioural changes may contribute to less EMS evaluations for chest pain or STEMIs, since patients may be reluctant to seek medical contact as they fear being infected or are unwilling to burden the healthcare system even further.

Other studies have reported an increase in OHCA during COVID-19 lockdowns [2–4]. Marijon et al. suggested that OHCA occurring in patients with respiratory or cardiovascular complications of COVID-19 as well as OHCA related to advanced cardiac injury in late STEMI presenters might explain the observed OHCA increase [4]. In the current study, the incidence of OHCA remained unchanged. During the COVID-19 lockdown period, however, there was a trend towards a different cause of OHCA, with less patients with a shockable rhythm upon arrival by the EMS and more patients with cardiac complaints shortly before the occurrence of OHCA. Conceptually, this may concern patients who were reluctant to seek medical help in an earlier phase of acute myocardial infarction. Combined with the fact that the total number of OHCA patients in our study was relatively small and that the number of COVID-19 patients in the Netherlands was relatively low compared with that in Italy and France, this might explain that we only saw a shift in causes of OHCA rather than an increase in OHCA incidence. Of note, the aetiologies were defined through case-by-case evaluation of the EMS reports—and not through postmortem pathology, the golden standard—and are thus subjective.

### Strengths and limitations

When interpreting the results of the current study, its strengths and limitations should be taken into account. The most important strength is the use of the AmbuSuite database, which contains prospectively collected data of all ambulance transports in Hollands-Midden, a Dutch ‘security region’ with over 800,000 inhabitants.

Unfortunately, no data from other regions were available. Further research is needed to conclude if these findings are similar in, for example, regions with less air pollution. Furthermore, this database does not provide insight into people with chest pain who decide not to contact the EMS. In addition, it does not contain individual outcome data. Therefore, it was not possible to analyse whether clinical outcomes of chest pain patients evaluated by the EMS was better or worse during the COVID-19 lockdown period.

## Conclusion

We showed a significant decrease in the number of patients with chest pain evaluated by the EMS. This was paralleled by a reduction in the incidence of STEMI, while the incidence of OHCA remained unchanged (see Fig. 4 in Electronic Supplementary Material). While the reason for the decrease in chest pain and STEMI incidence is not entirely clear, there are multiple possible factors. A decrease in physical exertion, a dramatic decrease in air pollution and reluctance to contact medical authorities during the COVID-19 lockdown could have played a role in the reduced number of ambulance transports for chest pain and STEMI. Alerting the public to the importance of contacting the EMS in case of suspected cardiac complaints may help to reduce the secondary health damage in case of possible future lockdowns.

## Supplementary Information

Fig 4. Graphic abstract showing the results from this study.
