# Supplementary figures and images for: Emergency medical services evaluations for chest pain during first COVID-19 lockdown in Hollands-Midden, the Netherlands
Source: Neth Heart J. 2021 Feb 18;29(4):224–9. doi: 10.1007/s12471-021-01545-y (PMC7890775; doi:10.1007/s12471-021-01545-y)

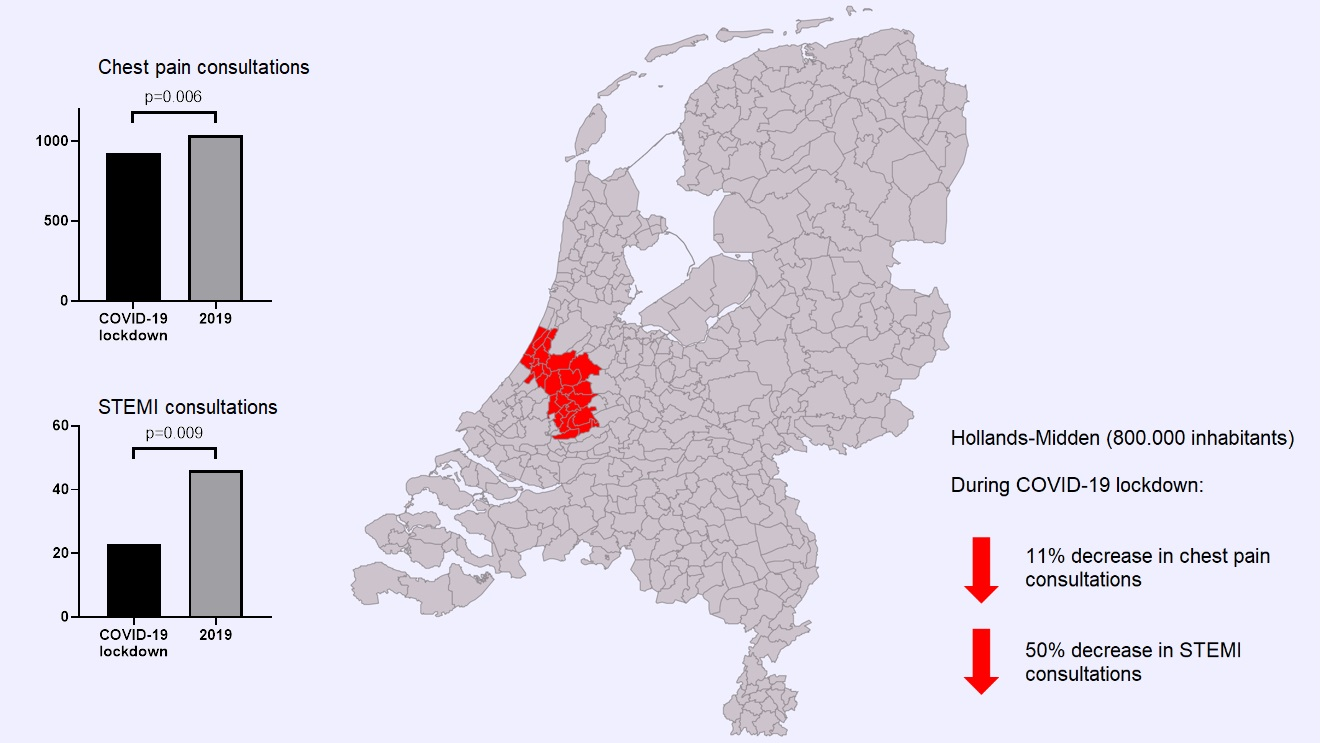

Supplement: Supplementary file 1 — Fig 4. Graphic abstract showing the results from this study. [file 12471_2021_1545_MOESM1_ESM.tif]
